# Event-Driven Data Gathering in Pure Asynchronous Multi-Hop Underwater Acoustic Sensor Networks

**DOI:** 10.3390/s20051407

**Published:** 2020-03-04

**Authors:** Sara Blanc

**Affiliations:** ITACA-UPV, Universitat Politècnica de València, camí de Vera s/n, 46022 València, Spain; sablacla@disca.upv.es

**Keywords:** underwater sensor networks, asynchronous systems, acoustic, wake-up tone, data gathering, gliders, handshaking, MAC protocols, polling, NS-3 simulation

## Abstract

In underwater acoustic modem design, pure asynchrony can contribute to improved wake-up coordination, thus avoiding energy-inefficient synchronization mechanisms. Nodes are designed with a pre-receptor and an acoustically adapted Radio Frequency Identification system, which wakes up the node when it receives an external tone. The facts that no synchronism protocol is necessary and that the time between waking up and packet reception is narrow make pure asynchronism highly efficient for energy saving. However, handshaking in the Medium Control Access layer must be adapted to maintain the premise of pure asynchronism. This paper explores different models to carry out this type of adaptation, comparing them via simulation in ns-3. Moreover, because energy saving is highly important to data gathering driven by underwater vehicles, where nodes can spend long periods without connection, this paper is focused on multi-hop topologies. When a vehicle appears in a 3D scenario, it is expected to gather as much information as possible in the minimum amount of time. Vehicle appearance is the event that triggers the gathering process, not only from the nearest nodes but from every node in the 3D volume. Therefore, this paper assumes, as a requirement, a topology of at least three hops. The results show that classic handshaking will perform better than tone reservation because hidden nodes annulate the positive effect of channel reservation. However, in highly dense networks, a combination model with polling will shorten the gathering time.

## 1. Introduction

Our seas and oceans need us to take care of them. The observation and control of our blue spaces is very important if we want to achieve a balance between maritime exploitation and the preservation of natural spaces and water quality.

In the last two decades, great efforts have been made to develop more effective and robust technology, for both measurement instruments and autonomous underwater vehicles. However, designing and building complete information systems remains a challenge in marine environments. In addition to the known difficulties for the transmission of signals in water, it is important to remember that humans live on land. Therefore, a valid information system for water observation and control must include a means of transferring information out of the water. Sea–land data transference seeks to explore both suitable underwater protocols and land access, taking into account water channel problems [1] and propagation delays [2].

Future information systems should combine sea–air communication. For example, similar to Internet of Things (IoT) land systems, such devices or sensors could remain connected to cloud processing systems continuously. Thus, sea infrastructure should include surface buoys responsible for the Internet access (Gateways). However, this is not the only possible model. It is also possible to design a discontinuous communication model that uses autonomous vehicles or vessels to link both water and air channels. Depending on the scenario, continuous or discontinuous communication will be easy to deploy to facilitate data transmission.

For a discontinuous model, data collection is discrete and triggered by an event (Event-Driven)—for example, the appearance of a vehicle able to gather data from a network while avoiding physical rescue of the submerged instruments.

For data gathering, the underwater network paths are built according to the location of the collector or sink that established a link with the vehicle. We must assume that sensor devices deployed in the water channel continue collecting data for long periods of time so that these data can be transmitted smoothly to the vehicle when necessary. Therefore, the first difference with a continuous model is traffic density. In a continuous model, each sensor deployed underwater collects information and retransmits it to the sink, usually with a low frequency. Traffic density is considered to be low but constant, and the probability of collision is also low. However, in a discontinuous model, the vehicle is not always available. Therefore, the period of time during which data can be retransmitted to the vehicle is limited and is triggered at the very moment that the vehicle establishes a valid link with one of the deployed nodes that will serve as the sink. That increases the traffic density, which will be high at the time of gathering.

Some recent works have explored vehicle-based data gathering [3,4,5,6,7,8,9,10,11]. These studies focused on routing protocols and explored alternatives to determine the node or nodes that will serve as a sink between the network and the vehicle. These studies give a clear idea of the potential use of autonomous vehicles to collect information from a permanently deployed underwater network.

In underwater vehicles, gliders have advantages over other Autonomous Underwater Vehicles (AUV) because of their resistance and capacity to carry out long surveillance campaigns. However, the uncertainty in the navigation route of a glider in the open sea is a significant handicap. Sink nodes should, therefore, be based on discovery, selected among those with better range and a higher link quality to the glider.

Once the link between the vehicle and sink node is established, the network paths are built. Some works, such as the study in [12], consider dynamic path building to be more resilient to the void problem due to the fact that paths are rebuilt periodically. We consider this advantage in the present proposal. These paths are ephemeral; they will be built at the data gathering time and broken up after finishing data collection.

Thus, given a defined water volume, when a vehicle establishes its link with a sink, the building of network paths and data transmission from nodes to the sink are triggered. These events are asynchronous. Thus, physical asynchronous mechanisms are more suitable for working with discontinuous networks because of their ability to simply start transmission of messages at the very moment a link is established with the vehicle.

Asynchronous wake-up mechanisms present clear energy saving advantages compared with synchronous networks [13]. Nodes do not need to stay synchronized, as they will wake up due to an event; this is called Acoustic-Triggered for Wake-Up (AT-WU) or a wake-up tone. This tone is received just before receiving a message. These wake-up tones can be broadcast or unicast and are based on an adaptation of terrestrial Radio-Frequency Identification (RFID) technology to acoustic networks [14]. This adaptation provides nodes with a low range of over 300 meters but low energy consumption [15].

Thus, this proposal is focused on a discontinuous scenario with high traffic peaks, which uses AT-WU. Moreover, network nodes will remain without communication for long periods of time, thereby avoiding energy consumption overhead for synchronization purposes. As this scenario presents a clear advantage in terms of energy savings of the nodes, this paper explores how Medium Access Control (MAC) protocols could be adapted within multi hop networks.

MAC protocols in underwater scenarios present important differences with respect to their terrestrial counterparts. The effect of the channel delays in acoustic communication is especially significant. Among the solutions based on Carrier-Sense Multiple Access (CSMA), several protocols introduce coordination mechanisms between the receiver and the transmitter, especially for highly dense networks. Based on the adaptation of MACA-P [16] to multi-hop sub-aquatic networks with MACA-U [17], there are many examples that explore multi-hop topologies. Among contention-based solutions, many handshaking protocols have adapted the classic Request-To-Send/Clear-To-Send (RTS/CTS) as an underwater scheme [18,19]. As an alternative, T-Lohi [20] used reservation tones instead of RTS/CTS control packets. Although T-Lohi was designed for asynchronous networks, it was only applied to one-hop topologies. Finally, in addition to handshaking, collisions have been successfully reduced in some studies by introducing scheduling mechanisms [21,22] or polling [23,24]. This paper, as novelty, explores the adaption of AT-WU mechanisms while attending to the above three groups: classic handshaking, tone reservation, and scheduling by polling.

## 2. Materials and Methods

### 2.1. The Routing Protocol

Any multi-hop network solution requires a routing protocol. Because energy consumption is critical in underwater networks, our proposal focuses on building network paths while considering balanced energy effort distribution in the relay selection.

Location free routing protocols do not assume location knowledge, but they do require a keystone parameter to build the network paths. Building is triggered by a sink in a flooding style, while forwarding relays are decided as a function of their distance to the sink. Depth or hops number are also combined with residual energy, link quality, and network density in the relay forwarding decision process.

Depth is a parameter relative to surface data gathering throughout the water column. Thus, this premise is not valid for glider gathering, whose angle and depth varies in a 3D region as a function of its navigation wave trajectory. However, path distance or hop numbers are feasible decision parameters.

The number of hops to the sink can be transmitted as part of the path building frames without computational overhead. This has been widely used in multi-hop routing protocols (assuming their calculation is a non-time-consuming task). Many location free routing algorithms have been designed in a hop-by-hop decision style, with good results using only parameters relevant to the current hop, such as the neighbors’ link quality or the minimum distance among potential relays, as long as the transmission power can be adjusted to save energy. Moreover, recent examples of routing protocols [25] have considered the residual energy of potential relays to decide which one will forward its data through the sink [26,27,28,29]. The objective is to select the relay with the greatest residual energy.

Therefore, once the sink is established, the following premises for handshaking will be taken into account: (i) Any node in the network can be a relay for another node that is +1 hops from it; (ii) relays are decided hop-by-hop; (iii) a sender node chooses its relay by itself based on the residual energy of the potential relay nodes.

The sink begins the network with a Path-Builder (PB) control packet, with its hop number initialized to zero and its residual energy. This event triggers network building and DATA packets transmission. Nodes in the sink range will receive the PB control packet and try to send their payload to the sink. Any node in range will hear the channel via the Channel Collision Avoidance (CCA) noise detection mechanism. Before sending (carrier sensing) its payload. After transmitting its payload to the sink, a node that is free of its own payload will broadcast a new PB control packet, thus becoming a potential relay. Thus, PB transmissions and payload transmissions occur simultaneously.

Data transmission is a function of *C*, Δ, and *δ*, where *C* is the 3D underwater cube (length, weight, height), Δ the maximum number of nodes in range to a hop-relay with upgraded data, and *δ* is the overall network payload density in *C*.

Network path building needs to be cross-combined with a MAC protocol. The long propagation delays underwater make the handshaking-based protocols inefficient since there are errors present underwater that do not happen on land scenarios. However, the disadvantages of handshaking underwater can be mitigated by limiting the 3D volume. For example, assuming that a glider reaches the maximum depth (height) every Nm^2^ in the navigation path (2D projection: length, weight), the maximum 3D volume can be set by attending to these three parameters. The knowledge of the maximum propagation delay in *C* will serve to limit the maximum number of hops, thereby making the occupation of the channel more efficient in short ranges.

This proposal joins the well-known MACAW [30] protocol with the following rules:

- When a relay receives a DATA packet, the relay prioritizes forwarding that packet to the sink. If the relay has a chance to win the channel, it will send an RTS packet to its own relay or sink. However, before winning the channel the relay may possibly receive an RTS packet addressed to it. In that case, the relay will reject this last RTS packet. This strategy will accelerate the arrival of packets to the sink but increase the energy effort, as data serialization does not occur. *δ_i_* is the hop density, which is the number of packets of data to be transmitted to the next hop. The maximum *δ* is given by expression (1):
  (1) $$\delta =\;\overset{i=\Delta }{\underset{i=0}{\sum }}i\delta_{i}$$
- After receiving one or several PB packets, a node must decide its relay node. Each node maintains a relay table. The PB packets include the hop number to the sink and the relay’s current residual energy. Given *C*, a greater Δ indicates that more possibilities exist to shorten the transmission beam towards the sink. However, each hop involves a new necessary competence to win the channel with an additional delay. Thus, this proposal prioritizes achieving the minimum number of hops.
- The relay choice priority is dynamically updated based on residual energy. CTS and Acknowledge (ACK) packets include the residual energy of the packet sender. Receivers will update their relay-table and the priority list. Thus, the CTS and ACK packets are always broadcast.
- After sending a PB packet, the new relay remains sleeping. The neighbor *i*, before choosing a relay, waits until a maximum deadline time *ρ_i_* in order to receive more PB packets from its *n_i_* neighbors, where $\pi_{max\_hop\_prop}$ is the maximum one-way prorogation time per hop.
  (2) $$\rho_{i}=\;\pi_{max\_hop\_prop}\;\times \left(n_{i}-1\right)+\left(backoff_{max}\;\times \left(n_{i}-1\right)\right)$$

### 2.2. Basics on Handshaking in Asynchronous Multi-Hop UANs (WU-RTS/CTS/DS)

AT-WU external systems reduce the modem power consumption near the ideal [15]. With an AT-WU system, the node remains sleeping until it detects a pre-defined Wake-Up (WU) signal. Differences to radio frequency communication are especially relevant for the design and development of reliable transducer adapters. Instead of antennas, underwater acoustic communication needs electro-acoustic transducers, which are more expensive. In order to reduce costs, a design is desirable with only one transducer per modem. However, AT-WU tones are modulated following an On-Off Keying (OOK) modulation scheme, while packets follow Frequency-Shift Keying (FSK) modulation. In [31], both signals OOK and FSK signals are transmitted and received by the same transducer. Thus, at the MAC level, both are treated as packets. However, AT-WU does not contain network information, only a wake-up pattern.

**Figure 1:**
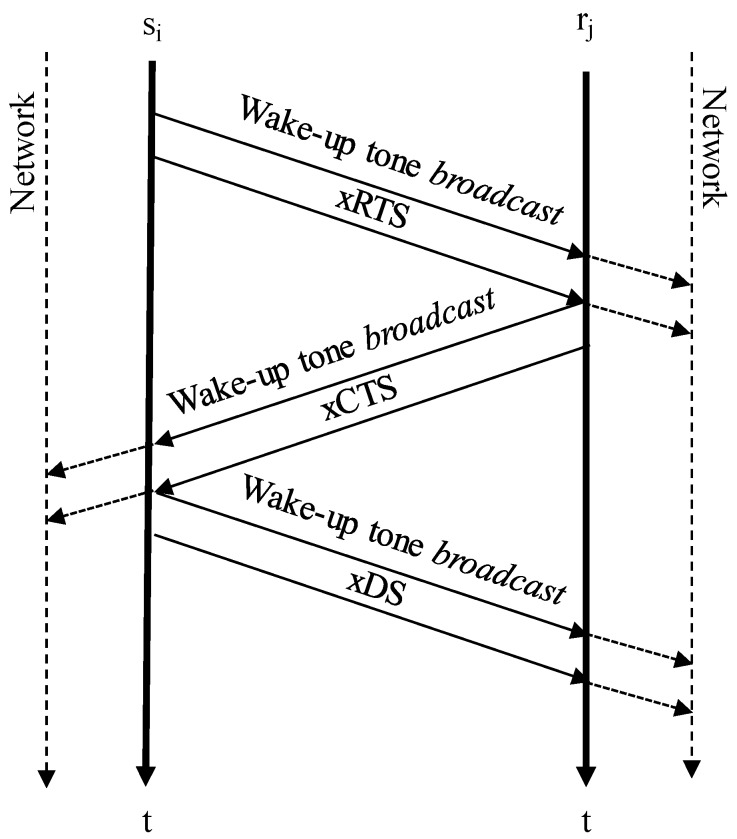
WU-RTS/CTS/DS handshaking with an AT-WU modem.

Figure 1 shows the overall concept of a contention-based non-slotted persistent protocol. In addition to RTS/CTS handshaking, the MACAW protocol introduced the Data Sending (DS) control packet for multi-hop networks [30]. DS packets act as a CTS packet for nodes that are out of the relay range. The sender, after receiving the real CTS, extends the contention effect to its own neighbors by sending the DS packet.

The reception of a PB, RTS, CTS, or DS packet is considered an event. Events switch the node from sleeping to active. Assuming *s_i_* is a sender node and *r_j_* is a valid relay for *s_i_*, *r_j_* is woken up by a wake-up tone just before receiving the RTS packet. The wake-up tone and the control packet are sent in a burst style with a minimum delay between both frames, known as the Inter Tone Gap (ITG). After RTS transmission, *s_i_* switches into sleep mode. Senders wait, sleeping, for a CTS response; after a maximum timeout with no answer, the senders go back to sleep for a backoff period before competing again for the channel.

After receiving an RTS packet, the relay will broadcast a wake-up tone and an xCTS (x means broadcast). Thus, CTS is heard by *s_i_* and by any other node in range of *r_j_*. Control packets include:

- Destination address: Packets are broadcast or unicast; in which case, the destination address matches the relay or sink address.
- Source address: The packet owner address.
- Relay address: The CTS and DS packets specify the relay in the contention window.
- Reserve address: The CTS and DS packets specify the sender owner of the contention window.
- Residual energy: PB, CTS, and ACK frames include the packet sender’s residual energy.
- DATA size: This is needed to calculate the Network Allocation Vector (NAV) time of the contention window [32].
- Hop number: The number of hops from the packet sender to the sink.
- Sequence number: The DATA packet sequence.
- Frame ID: Identifies the type of the frame.

During the contention window, no other node in range of *r_j_* should try to win the channel. Similar to an xCTS, the xDS control packet notes the channel reservation to nodes in range of *s_i_*. DS has an additional goal. Any node in the network is both a sender and a relay. When a relay holds data, it will try to retransmit those data as soon as possible. However, during the same time that this relay tries to send data, it could still receive an RTS for any other sender in range. The DS will protect its contention window as a sender from receptions as a relay.

**Figure 2:**
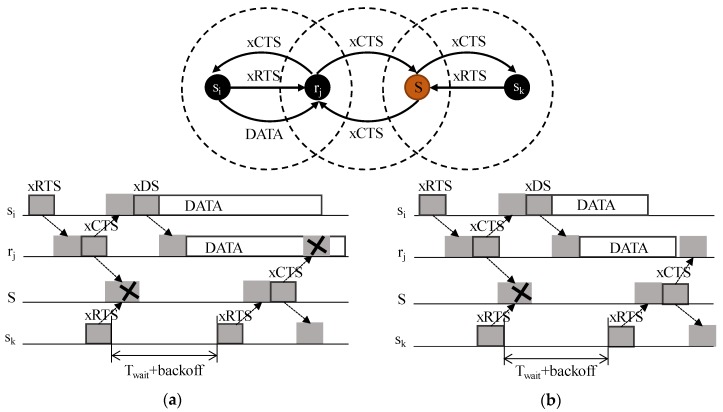
Multi-hop scenario without wake-up tones; (**a**) A long data frame causes CTS collisions; (**b**) A short data frame avoids CTS collisions.

However, this contention-based protocol is not exempt of collisions in the underwater channel. Figure 2 shows a multi-hop configuration. The backoff time is always preceded by a *T_wait_* time that depends on a previous action. This time takes into account the delays of both channel propagation and packet processing. As shown in Figure 2, the xCTS transmitted by relay *r_j_* and the RTS packet transmitted by sender *s_k_* cause a collision in sink *S*, which will not receive the CTS nor the RTS packets. Due to this collision, sender *s_k_* will not receive any answer to its RTS packet and will try again after the backoff time. In the case of long DATA frames, the waiting time will not be long enough to avoid the collision shown in Figure 2a. To reduce the probability of this scenario and to shorten the DATA frame size as in Figure 2b, relays forward their DATA packets independently without data treatment or serialization. This makes the size of the payload constant from the source to the sink.

**Figure 3:**
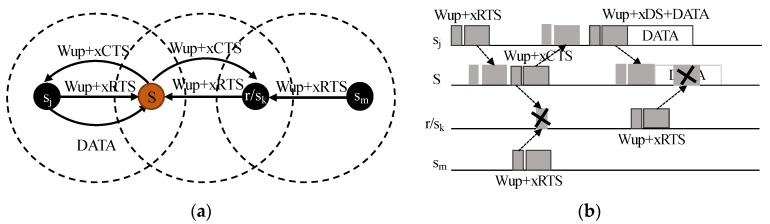
Handshaking multi-hop scenario adding asynchronous wake-up tones (**a**) and a conflicting example (**b**).

Asynchrony introduces an inherent problem. The premise of an asynchronous node is that it will remain sleeping. Thus, an asynchronous node will save energy, which is a clear advantage. However, a node is capable of hearing a CTS or a DS packet only when woken up before the reception of this packet. Figure 3a shows the multi-hop scenario with Wup tones and a conflicting scenario in Figure 3b. Wake-up tones broadcast by sink *S* and sender *s_m_* collide in node *r/s_k_*. The relay does not receive the CTS packet and, therefore, does not comply with the contention window. The higher the density of the network, the more collisions will occur.

### 2.3. Introducing Tone-Competitions to Win the Channel (T-WU-RTS/CTS/DS)

Most of the collisions occur during channel competition. Thus, a variation can be added to the model above (WU-RTS/CTS/DS). Similar to T-Lohi [20], which used tones as a contention mechanism, T-WU-RTS/CTS/DS extends tones to a multi-hop scenario combined with handshaking.

Choosing the model in [31], the wake-up tones consist of a bit string equivalent to the receiver ID. The tone does not contain information about its own identity. The usefulness of wake-up tones is that they wake up a receiver just before it receives a packet. However, in a T-WU-RTS/CTS/DS variation, instead of a sequence featuring a wake-up tone plus a packet, the node broadcasts only a wake-up tone in order to reserve the channel. In case of failure, the energy effort in transmitting the RTS packet, which is completely useless, will be saved (node *s_m_* in Figure 3).

Assuming an omnidirectional modem, the nodes behave as senders and relays in the network. Wake-up tones are significantly shorter than packets. Therefore, the probability of success in detecting whether the channel is free or busy by means of CCA mechanisms depends on the time during which the CCA is active. Algorithm 1 explains the behavior of one node, which seeks to win the channel. In Algorithm 1, the CCA is active according to expression (3): (i) before sending a wake-up tone during the backoff time in expression (4), where *RTS_post_* is the number of failed RTS attempts of the sender; and (ii) during competition time, where $t_{wup\_frame}$ is the time needed to transmit a wake-up tone. Thus, before sending an RTS packet, the node competes for channel for $t_{competition}$ time in expression (5). Expression (5) is based on aUT-Lohi [20] for asynchronous and aggressive networks. CCA being active means that in addition to the reception of wake-up tones, channel noise up to a certain SINR (Signal to Interference plus Noise Ratio) will also be interpreted as the existence of tones in the channel.

(3) $$t_{CCA_{active}=\;}t_{backoff_{i}}+\;t_{competition\;}$$

(4) $$t_{backoff_{i}}=random\;\left[0,1\right]\times \pi_{\max\_hop\_prop}\;\times 2^{\left(RTSpost_{i}+1\right)}$$

(5) $$t_{competition\;}=t_{\mathrm{avg}\_ToA}+t_{2}\;=\;\pi_{\max\_hop\_prop}+t_{wup_{frame}}$$

**Figure 4:**
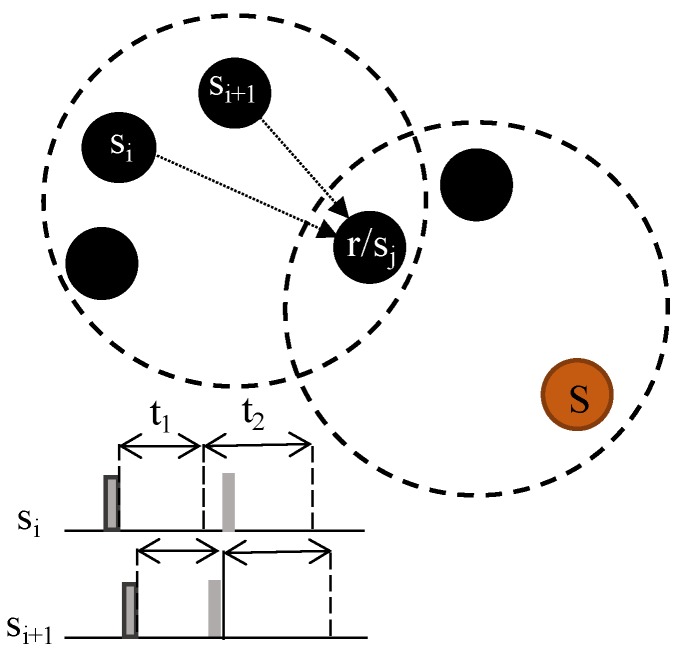
Wake-up reception in two neighboring nodes.

Given a subset of neighbor nodes with an equal hop number, the average time of arrival among them is defined as $t_{\mathrm{avg}\_ToA}$. *s_i_* and *s_i_*_+1_ are two equidistant nodes competing for the channel, and *t*_1_ in Figure 4 is set to the $t_{\mathrm{avg}\_ToA}$. The *s_i_* tone is received by *s_i_*_+1_ in *t*_1_, which means that *s_i_*_+1_ loses the channel.

**Figure 5:**
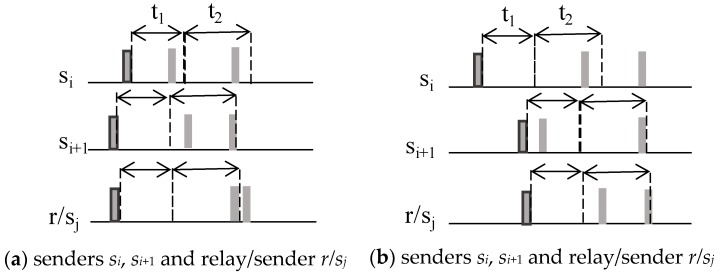

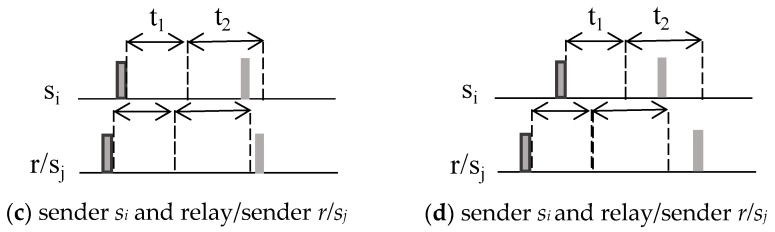
Special cases not covered by Algorithm 1.

Channel competition only concerns wake-up tones. Figure 5 shows some cases outside Algorithm 1. Between *s_i_* and *s_i_*_+1_, $t_{\mathrm{avg}\_ToA}\;<\;\pi_{\max\_hop\_prop}$, while between *s_i_* or *s_i_*_+1_ and *r/s_j_*, $t_{\mathrm{avg}\_ToA}$ is near $\pi_{\max\_hop\_prop}.$

In case (a), *s_i_*_+1_ wins the channel; in case (c), *s_i_* wins the channel. The relay *r/s_j_* in sender mode (*s_j_*) resigns due to the tone detected just after $t_{competition}$.

In cases (b) and (d), both nodes win the channel, and handshaking functions as in WU-RTS/CTS/DS.

During $t_{1}$, if a node detects a wake-up tone or noise in the channel, it switches to sleep for the time in expression (6). Moreover, after sending an RTS packet, the sender goes to sleep for a maximum of $t_{reserved}$ and will only wake up when receiving a CTS or a new wake-up tone from a neighbor node.

(6) $$t_{reserved\;}=\;(3\;\times t_{control\;packet})+\left(2\;\times \;(\pi_{max\_hop\_prop}+t_{wup_{frame}}\right)$$

$t_{control\;packet}$ in expression (6) refers to RTS, CTS, and DS. The control frames are at least as long as the $t_{avg\_ToA}$ time among nodes, which competes for the same relay.

Because channel competition is done prior to the RTS transmission, the wake-up tone that precedes any RTS packet is sent in unicast mode. Thus, the neighboring nodes remain sleeping and save energy.

**Algorithm 1.** Tone-competition internal algorithm for senders.**Init**: Sender *s_i_* waits for a backoff time in expression (4), win = *false*While $0\leq t_{0}\leq t_{backoff\_i}$ loopDuring $t_{0}$, if the sender *s_i_* receives a wake-up tone or detects noise in the channel then go to sleep $t_{reserved}$; ***go to step 10***; Sender *s_i_* sends a wake-up tone;While $0\leq t_{1}\leq t_{avg\_ToA}$ loopDuring $t_{1},$ if sender *s_i_* receives a wake-up tone or detects noise in the channel then complete $t_{competition}$ and go to sleep $t_{reserved}$; ***go to step 10***;win = *true*;While $0\leq t_{2}\leq \pi_{max\_hop\_prop}+t_{{wup}_{frame}}$ loopDuring $t_{2}$, if the sender *s_i_* receives a wake-up tone or detects noise in the channel, then **discard**;If the channel is not busy, send a wake-up tone to *r_j_*, else go to sleep $t_{reserved}$*; **go to step 10***;Wait $t_{ITG}$If the channel is not busy, send an RTS packet to *r_j_*,Go to sleep $t_{reserved}$; wait CTSIdle mode, listen to the channel during: $t_{ITG}$In the case of CTS, process according to the handshaking protocol; ***break***;In the case of no CTS, go to sleep $t_{reserved}$; ***back to step 1***;

### 2.4. NAV Asynchronous Adjustment and List Energy Update

By receiving a CTS packet, a node will remain sleeping for the time outlined in expression (7) before any new competition attempt. This contention window will be adjusted by the reception of an DS packet, as in expression (8). However, during this contention window, the node can be woken up by a broadcast wake-up tone. Due to the spatial–temporal uncertainty in the underwater channel, with *s_i_* as a neighbor node of the sender node *s_j_* and the owner of a contention window, the *tcw_max_i_* in expressions (7) and (8) is oversized. To reduce the NAV time, the contention window can be closed abruptly when receiving a closure packet. Closure packets are presented in the form of Path-Builder (PB) or Acknowledge (ACK) packets. Both PB and ACK are always broadcast and preceded by a wake-up tone. Moreover, they contain the residual energy of this packet sender, allowing the receivers to update this value in the relay-list. Therefore, only DATA packets are not preceded by any wake-up tone.

(7) $$tcw\_max_{i}=t_{DS}+t_{DATA}+\;t_{ACK}+\left(3\times \;\pi_{max\_hop\_prop}\right)+\left(2\times \;t_{wup_{frame}}\right)+\left(2\times \;t_{ITG}\right)$$

(8) $$tcw\_max_{i}=t_{DATA}+\;t_{ACK}+\left(2\times \;\pi_{max\_hop\_prop}\right)+\;t_{wup_{frame}}+\left(2\times \;t_{ITG}\right)$$

### 2.5. Introducing the Polling Request (WU-POLL)

Many MAC protocols assume pre-stabilised paths in the network because of several advantages in shortening trace beams. This implies that only a subset of network nodes would be relays of a known and pre-defined set of +1 hop nodes.

Although building the paths during the gathering time would better solve the void problem, such an “on-time-building” would increase the traffic of the network due to the transmission of as many PB packets as there are nodes in the network.

We can compare the classic WU-RTS/CTS/DS and Tone-competition T-WU-RTS/CTS/DS with a polling approach.

Some rules should be defined for the polling process defined in Algorithm 2:

- ACK are unicast transmissions with no wake-up tones. Thus, energy is updated by a new type of packet called PE (Polling End). PE packets are broadcast at the polling slot end and contain the remaining energy of the relay.
- xCTS and xDS packets are useful only in “relay” mode to calculate the NAV value in expressions (7) and (8). A node in “sender” mode does not need to calculate the contention window. After sending an RTS packet, it switches to sleep and only wakes up with a new tone.
- RTS wake-up tones are broadcast transmissions (xRTS). The RTS reception of a neighbor node resets and updates the backoff timer. The *T_wait_* before the backoff time is $\pi_{max\_hop\_prop}$.
- PE reception switches the dual nodes into “relay” mode only in the case of no pending packets.
- To adapt polling to multi-hop topologies, a new packet called RQ (ReQuest) is introduced. RQ packets are sent by relays with pending data to request a polling process over its own −1 hop relay.

**Algorithm 2.** Polling algorithm.**Init:** Relay *r_j_* or *S* send a PB packetWait $t_{backoffmax}+\pi_{max\_hop\_prop}+t_{{wup}_{frame}}$If the RTS packet is received, then add to the list and go ***back to step 1***;  else ***go to step 3***;While list_size > 0 start the polling process to the RST list, loopSend PE packet, sleep, ***break***;**Init**: Sender *s_i_* receives PB packetWaits for $\rho_{i}$ in expression (2)Waits for the backoff time in expression (4)If the channel is not busy, send a wake-up tone and xRTS to *r_j_*, else ***back to step 2***;Go to sleepReceive the CTS packet and wake-up; process according to xDS/DATA/ACKSleep and wait for PE to update relay energy valueIn case of dual-mode, it switches to “relay” mode and sends the PB packet and ***back to step 1***; else ***break***;**Next:** Relay *r_j_* or *S*    8. If packets pending, send RQ to *r_j_*_−1_ or *S*    9. Go to sleep    10. If PB is received, ***back to step 1***;

## 3. Results

As stated above, handshaking in AT-WU asynchronous networks can be implemented following three variations or models:

1. (WU-RTS/CTS/DS), which adapts the classical RTS/CTS/DS schedule by adding the AT-WU tone to any control packet transmission. RTS/CTS/DS and ACK packets are broadcast transmissions, while both PB and ACK include the residual energy value of the relay. Sender nodes will, therefore, select their relays depending on the minimum hop number and residual energies.
2. (T-WU-RTS/CTS/DS), which adds tone-competition previously to RTS packet sending. It tries to reduce the number of collisions due to RTS packets. RTS packets are transmitted in unicast mode. CTS/DS and ACK packets are broadcast, while both PB and ACK include the residual energy value of the relay. Sender nodes will, therefore, select their relays depending on the minimum hop number and residual energies.
3. (WU-POLL) is a mixed model with handshaking and polling. It assumes that network relays are pre-defined. However, channel certain competition is already maintained. When a relay sends a PB packet, the nodes can respond with an RTS packet if they are still alive with pending data. The relay receives and stores the RTS list to conduct a polling stage just after receiving the last RTS. The polling slot is explicitly finished with a PE packet to allow both updating the residual energy value of the relay and as a token to force a mode change in dual-nodes, which switch into relay mode. Any relay in the network repeats the same process to receive the data from its +1 hop senders. In order to request a new polling stage to a -1 hop relay, the model introduces RQ packets. When a sleeping relay receives a RQ, it sends a PB in order to receive an RTS packet from any node in range with pending data. The CTS and DS packets are broadcast transmissions to avoid collisions due to RQ packets.

Simulations to compare these three models have been carried out in an ns-3 simulator [33] using an Underwater Acoustic Network (UAN) model and Thorp as an acoustic propagation path-loss model [33,34,35].

The energy consumption values for both OOK and FSK transmissions correspond with the values in Table 1. In a range of 150 meters, the wake-up tone of the system lasts only up to 10% of the $\pi_{\max\_hop\_prop}$.

**Figure 6:**
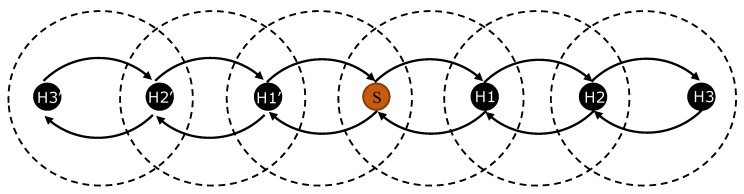
Multi-hop network topology abstraction.

In order to compare the above defined approaches, the models have been tested in the multi-hop scenario shown in Figure 6, which represents a 3D underwater cube (*C* = (900 m, 30 m, 30 m)). Comparisons are conducted for both time and energy. H1 and H1′ represent a subset of nodes with Δ {3, 4, 5, 6, 7} in range with *S* and hop = 1. Similarly, H2 and H2′ represent a subset of hop = 2, and H3 and H3′ represent a subset of hop = 3. Δ_H1_ = Δ_H1__′_ = Δ_H2_ = Δ_H2__′_ = Δ_H3_ = Δ_H3_.

### 3.1. Network Transmission Time

Each node in the network has a pending DATA packet. The size of a DATA packet includes 20 bytes of payload for the sensor measures. The whole packet transmission time is ≥$\pi_{max\_hop\_prop}$.

**Figure 7:**
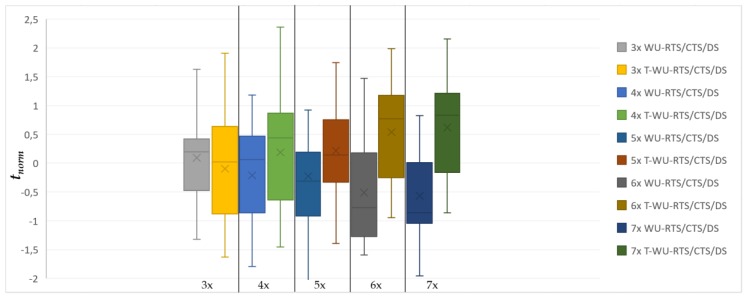
WU-RTS/CTS/DS and T-WU-RTS/CTS/DS comparison (X: Δx: number of nodes per the hop; Y: *t_norm_*).

**Figure 8:**
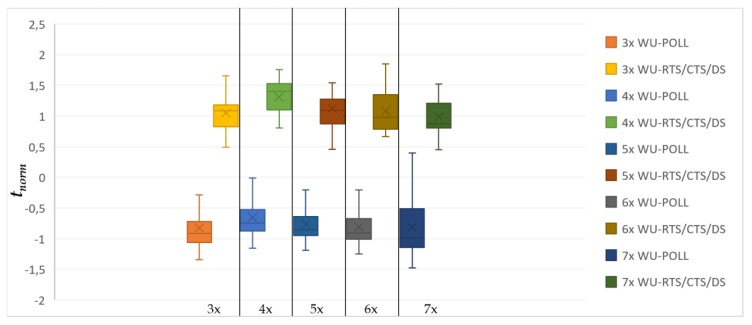
WU-RTS/CTS/DS and WU-POLL comparison (X: Δx: number of nodes per the hop; Y: *t_norm_*).

Figure 7 and Figure 8 show more than 500 simulations with different seeds. The number of nodes per hop in Figure 6 (number of nodes in range H_i_) is represented as Δ in the x-axis of both graphs.

The y-axis represents the normalized time observed in the simulation. This sample only includes simulations with a percentage of the expected messages delivered and successfully received by the sink of ≥98%.

**Figure 9:**
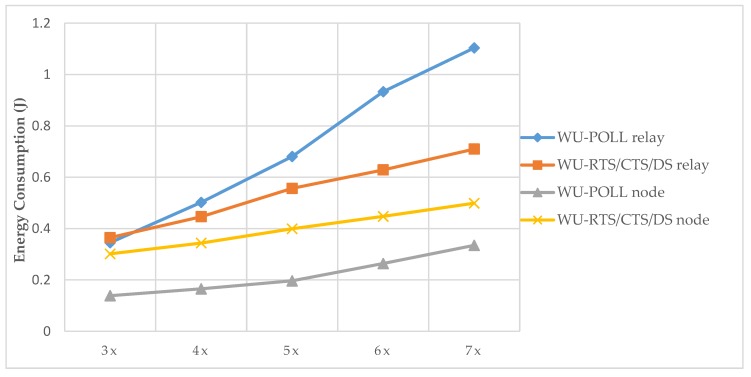
Relay energy consumption (X: Δx: number of nodes per the hop).

Nodes that do not receive the ACK will compete again. DATA packets are not serialized. However, in the WU-POLL model, a node that holds more than one DATA pending packet will send them sequentially during the same polling slot. Figure 8 and Figure 9 compare WU-RTS/CTS/DS with both T-WU-RTS/CTS/DS and WU-POLL.

With *δ* as the overall network payload density in *C*, Figure 7 and Figure 8 include five sampled densities {18, 24, 30, 36, 42}. Each sample in Figure 7 includes the observed time of both WU-RTS/CTS/DS and T-WU-RTS/CTS/DS, while Figure 8 includes the observed time of both WU-RTS/CTS/DS and WU-POLL.

The *normalized time* in the y-axis follows the expression (9):

(9) $$t_{norm}=\;\frac{t-\bar{x}}{\sigma }$$

where $\bar{x}$ is the mean value, and *σ* is the standard deviation per sample. Whisker plots are paired to compare the behavior of the models. The lower the mean value of the box, the better throughput the model presents.

In a multi-hop scenario, T-WU-RTS/CTS/DS is not efficient due to AT-WU tones collisions. Tone collisions pull back the tone reservation mechanisms, transforming the model into WU-RTS/CTS/DS normal behaviors. Thus, although the T-model could be more efficient with very few nodes per hop or a one-hop topology, it becomes a slower version of WU-RTS/CTS/DS as the number of nodes and hops increases. In Figure 7, we observe the clearly worse performance of the T-model with Δ = 5x density. Moreover, the T-model presents energy consumption that is usually greater than WU-RTS/CTS/DS—up to 0.5J in the worst case.

Figure 8 compares the WU-RTS/CTS/DS and WU-POLL models. Whisker plots are also paired to compare both behaviors. We observe that model WU-POLL performs better over time than the adapted classical handshaking model to any Δ. Figure 8 assumes only one relay per hop. It decreases the RQ competition. Thus, this figure shows the best case with a minimum number of relays. As the number of relays increases in the WU-POLL model, it converges to the WU-RTS/CTS/DS, thereby losing its sense of polling. Thus, this model performs better than the typical RTS/CTS/DS when the number of potential relays per hop is less than Δ.

### 3.2. Energy Effort

Figure 9 compares energetic relay effort of the WU-RTS/CTS/DS model and WU-POLL model with different hop sizes (Δx).

As shown in Figure 9, the WU-POLL relay consumes more energy because, during the polling slot, it remains awake. The difference between the WU-POLL model and the WU-RTS/CTS/DS model is that the relay remains longer in the IDLE and RX mode during polling. However, after adapting typical handshaking, the relay remains longer in SLEEP mode. The effort in TX mode, however, is equivalent in both models.

On the other hand, for non-relay nodes, the WU-POLL model is more conservative since the competition for the channel and the effort in forwarding failed RTS packets is reduced. In the case of WU-POLL, the original sense of the RTS packets is lost. The node sends a packet and switches to SLEEP mode without any control over the reception of the RTS packet. The effect we observed is that, as the number of nodes in the hop grows, more RTS packets collide in the relay. A mechanism exists to recover these lost packets by sending another PB packet as a response to an RQ. However, with the WU-RTS/CTS/DS model the competition for this channel after RQ increases, converging at a high density per hop.

## 4. Discussion

Classical RTS/CTS/DS handshaking has been adapted to purely asynchronous systems. Asynchronism is cross-layer built and considers both physical features and the MAC protocol. Adaptation is implemented and tested with and without taking advantage of tone competition. Tones last along only 10% of a channel’s acoustic distance and, hypothetically, would reduce energy efforts by reducing the number of failed RTS packets. However, although this premise is true for one-hop topologies or multi-hop topologies with a low Δ, the results showed worse performance than the simple RTS/CTS/DS adaptation in our scenario with more than 2 hops and significantly worse performance with 5 nodes per hop.

On the other hand, observing the results that compare the asynchronous adaptation of the classical RTS/CTS/DS handshaking model, WU-POLL, which mixes handshaking and polling, presents better time and energy performance. WU-POLL reduces data sending time to the sink which favors data-gathering throughput too.

However, WU-POLL presents two significant drawbacks. On the one hand, a sender node loses its control over channel reservation. After sending an RTS packet, the node does not know if the RTS packet has been received by the relay. Thus, it is necessary to implement additional rules to overcome this problem.

The sink/relay energy effort is higher than the adapted WU-RTS/CTS/DS model. During the polling process, the sink or relay remains awake to manage the channel accessing order. Thus, it turns off to sleep with a lower frequency than expected compared to the classical handshaking, where channel access management falls on the sender nodes.

Additionally, the WU-RTS/CTS/DS model presents some advantages. For example, because paths are built during the gathering time, this model better solves the void node problem. It is assumed that every node in the network will act as both a node and a relay. Thus, it is guaranteed that any node in a range with one or more nodes in the network will receive at least one PB packet to trigger its channel competition. Although isolation could occur due to relay failures, this model updates paths while attending to real accessibility, whereas WU-POLL needs to reduce channel competition to preserve its time and energy advantage by pre-defining fewer network relays than Δ. Thus, the loss of a relay has a high negative impact since it generates isolated areas.

In summary, this paper presents two efficient ways to adapt asynchronous modems (AT-WU) to maintain pure asynchronism at the MAC level. Both path building and data transference are event-triggered and driven coherently with event-driven discontinuous data gathering, using gliders as gateways to connect the sea and air channels.

However, future works should consider ways to reduce network traffic. For example, in the current version of the WU-RTS/CTS/DS and WU-POLL protocols, relays forward any packet received without checking redundancy. Simulations show that some ACK control packets in 6x and 7x are lost during transmission. Thus, data packets are retransmitted and re-forwarded by the relay, thereby increasing the traffic unnecessarily.

On the other hand, in WU-RTS/CTS/DS, throughput can also be affected depending on the forwarding approach. This proposal prioritizes retransmission to serialization. Data packets are forwarded as received instead of building new payloads serializing different data packets. Future work will analyze if this strategy is more effective than reducing the number of transmissions of a larger size.

Finally, both models, WU-RTS/CTS/DS and WU-POLL, could be enhanced with additional rules to save energy and reduce gathering time to take advantage of the time–space uncertainty [36] in the underwater channel. Moreover, the WU-POLL relay designation will be considered to maintain the balance between energy consumption and the minimum number of relays.

## Figures and Tables

**Table 1:** Energy consumption values corresponding with the modem described in [15].

Mode	Power
Sleep mode	3 µW
RX	24 mW
TX	120 mW
Idle mode	24 mW
WU RX	8.1 µW
WU TX	120 mW
